# Physiological status of House Sparrows (*Passer domesticus*) along an ozone pollution gradient

**DOI:** 10.1007/s10646-023-02632-z

**Published:** 2023-02-21

**Authors:** Concepción Salaberria, Carlos A. Chávez-Zichinelli, Isabel López-Rull, Marta C. Romano, Jorge E. Schondube

**Affiliations:** 1 Área de Biodiversidad, Departamento de Biología y Geología, Física y Química Inorgánica, Universidad Rey Juan Carlos, Calle Tulipán s/n, 28933 Móstoles, Madrid, España; 2 El Colegio de Puebla A.C, Avenida 41 Poniente 505, Col. Gabriel Pastor 1ra Secc, 72420 Puebla, Mexico; 3 Centro de Investigación y de Estudios Avanzados del Instituto Politécnico Nacional, 07360 Ciudad de México, México; 4 Instituto de Investigaciones en Ecosistemas y Sustentabilidad, Universidad Nacional Autónoma de México, Campus Morelia, Antigua Carretera a Pátzcuaro 8701, Colonia Ex Hacienda de San José de la Huerta, 58190 Morelia, Michoacán Mexico

**Keywords:** Air pollution, Bioindicator, Constitutive Innate Immunity, Corticosterone, House Sparrow, Urbanization

## Abstract

Mexico City is one of the most polluted cities in the world, and one in which air contamination is considered a public health threat. Numerous studies have related high concentrations of particulate matter and ozone to several respiratory and cardiovascular diseases and a higher human mortality risk. However, almost all of those studies have focused on human health outcomes, and the effects of anthropogenic air pollution on wildlife species is still poorly understood. In this study, we investigated the impacts of air pollution in the Mexico City Metropolitan Area (MCMA) on house sparrows (*Passer domesticus*). We assessed two physiological responses commonly used as biomarkers: stress response (the corticosterone concentration in feathers), and constitutive innate immune response (the concentration of both natural antibodies and lytic complement proteins), which are non-invasive techniques. We found a negative relationship between the ozone concentration and the natural antibodies response (*p* = 0.003). However, no relationship was found between the ozone concentration and the stress response or the complement system activity (*p* > 0.05). These results suggest that ozone concentrations in air pollution within MCMA may constrain the natural antibody response in the immune system of house sparrows. Our study shows, for the first time, the potential impact of ozone pollution on a wild species in the MCMA presenting the Nabs activity and the house sparrow as suitable indicators to assess the effect of air contamination on the songbirds.

## Introduction

Atmospheric pollution directly affects the health of human and wildlife species living in urbanized areas (review in Manisalidis et al. 2020). The negative effects of air pollution are more noticeable in megacities (metropolitan areas with a total population over ten million), where the emissions of atmospheric contaminants can be unprecedented in severity and extent (Molina and Molina, 2004). This is the case in the Mexico City Metropolitan Area (MCMA), which consists of Mexico City and its peri-urban area. The MCMA has a human population of more than 20 million, more than 5 million vehicles, and about 78,000 industrial buildings CONAPO, (2012). According to the World Health Organization (WHO), Mexico City was considered the most contaminated place in the world at the end of the 20th century, mainly due to a high number of toxic fuel vehicles and high industry activity (WHO, 1997). Following this report, several mitigating measures have been taken (including the creation of Mexico City´s environmental atmospheric monitoring agency), which have substantially reduced pollutants such as nitrogen oxides (NO_x_), carbon monoxide (CO), and sulfur dioxide (SO_2_). However, over the last two decades, both ozone (O_3_) and PM_10_ (particulate matter) still currently exceed the Mexican environmental standard guidelines (SEDEMA, 2016). The high levels of PM_10_ and O_3_ are considered a public health threat in Mexico City. Several studies conducted in MCMA have shown a positive relationship between the high concentrations of PM_10_ and O_3,_ and human mortality risk (Carbajal-Arroyo, et al. 2010; Romieu et al., 2012; Riojas-Rodriguez et al., 2014). MCMA residents are highly exposed to air pollution, and have been found to show signs of an early brain imbalance in genes involved in innate and adaptive immune responses (Calderón-Garcidueñas et al., 2012). Furthermore, Calderón-Garcidueñas et al. (2015) warned of the inflammatory effect of PM_10_ and O_3_ concentrations on the central nervous system in clinically healthy children living in the MCMA. Different studies on human health outcomes in other polluted locations have also found that ozone plays an important role as an inflammatory factor in airway diseases (Hiltermann et al.,^1998^, Schwela, 2000, Alexis et al., 2010, Oakes et al., 2013). Several studies have also shown a positive relationship between ozone concentration and levels of stress hormones in humans (Miller et al., 2016, Rajagopalan and Brook, 2016, Wang et al., 2022, Xia et al., 2022). Regardless of the concern about the potentially harmful effects of these and other air pollutants on human health outcomes (Héroux et al., 2015; Mannucc et al., 2015), knowledge of their consequences on wild animals is scarce, and most of the limited available evidence comes from birds. As in humans, some negative effects of pollutants have been found in wildlife (e.g Schilderman et al., 1997 for negative effects of heavy metals on DNA oxidative damage in pigeons; Gorriz et al. 1994 for negative effects of coal-fired power plants in the tracheal epithelium of passerine birds and small mammals; Herrera-Dueñas et al. 2014 for a comparison of oxidative damage in house sparrows between two zones differing in their pollution levels; Cruz-Martinez et al., 2015 for negative effects of industry pollutants on the immune response of tree swallows; North et al. 2017 for a relationship between traffic pollutants and morphology, immunity and oxidative stress in european starlings; Sanderfoot and Holloway 2017 for a review of adverse health impacts of different air pollutants on various bird species). For these reasons, birds have been used as a model species to evaluate the effect of atmospheric pollution on urban animal species, as they have been shown to be effective bioindicators due to their wide distribution, high sensitivity to pollutants, and their important role in urban ecosystem services (Furness, 1993; Brown et al., 1997; Swaileh and Sansur, 2006; Kekkonen et al., 2012).

Air pollution can cause stress and health problems to birds by activating different physiological processes, such as oxidative damage (Isaksson et al., 2017, Salmón et al., 2018), shortening telomeres (Salmón et al., 2016), and mobilization of heavy metals to feathers as a detoxification process (Chatelain et al. 2014). Pollutants can act as stressors by triggering a stress response, an evolved suite of physiological, hormonal, and behavioral responses which are exhibited and conserved across many vertebrate taxa (Wingfield and Ramenofsky, 1999; Romero, 2004). One of these responses in birds is secretion of the glucocorticoid corticosterone (Holmes and Phillips, 1976). The release of corticosterone is adaptive for birds in the short-term, as it facilitates survival of life-threatening challenges by elevating glucocorticoids to mobilize energy stores (Sapolsky et al., 2000), activating escape behavior (Wingfield and Romero, 2001), and diverting energy to self-maintenance via changes in behavior and physiology (Wingfield and Sapolsky, 2003). However, chronically elevated levels of this hormone have negative consequences to cognitive ability, growth, body condition and immune defense (Wingfield and Ramenofsky, 1999; Sapolsky et al., 2000). Besides the effects of glucocorticoids on immune responses, the immune system can also be directly affected by exposure to air pollutants. For example, solid particles and high rates of nitrogen oxides from the polluted air can cause a marked decrease in the number of pulmonary surfactant precursors, reducing the innate protection mechanisms of the lung, as has been reported for pigeons (*Columba livia*) living in the city of Madrid, Spain (Lorz and Lopez, 1997). Nestlings of tree swallows (*Tachycineta bicolor*) growing in air-polluted sites showed a reduction of the T cell response to the phytohemagglutinin skin test (Cruz-Martinez et al., 2015).

The high air pollution in MCMA could be affecting the health of the wild birds living there, however, there are no studies addressing this topic as all research to date has been focused on humans. For this reason, we evaluated the potential relationship between the concentration of air pollutants and two physiologic traits of the house sparrows: (1) stress (corticosterone concentration in feathers), and (2) constitutive innate immune response (concentration of both natural antibodies and lytic complement proteins). To achieve this, we used the natural air pollution gradient that exists in the MCMA. We predicted that both physiological responses would be decreased at sites with higher air pollution. Because other anthropogenic factors related to urbanization could be negatively affecting the physiology of birds, we also considered human population density, densities of houses and industrial complexes, as well as urban land-use at each of our sampling sites.

## Material and methods

### Study species

House sparrows are one of the world’s most broadly distributed species. They were introduced to North America in the 1850s from Europe and rapidly expanded to cover all of the United States and most of Canada and Mexico by the early 1900s (Grinnell, 1919). This species is closely associated with human activity and is highly abundant in urban landscapes. Several studies have shown that this species is sensitive to different stressors associated with urbanization level, which makes it a useful bioindicator. Individuals of this species have shown deficiencies in body condition (Liker et al., 2008; Bókony et al., 2010; Meillère et al., 2015), antioxidant capacity (Herrera-Dueñas et al., 2014), or feather quality (Meillère et al., 2017) in more urbanized habitats. This species also bioaccumulates persistent organic pollutants (Nossen et al., 2016) and heavy metals (Pinowski et al., 1994; Kekkonen et al., 2012; Millaku et al., 2014).

### Area characterization and measurements of air pollution

The MCMA is located on a high plateau more than 2000 m above sea level in the central part of Mexico. The MCMA consists of Mexico City, 59 municipalities of the State of Mexico, and 1 municipality from the State of Hidalgo. The MCMA has high concentrations of air pollutants, and because of its high altitude and low latitude, it receives relatively strong ultraviolet radiation that promotes photochemical reactions which generate O_3_ from precursor substances such as NO_x_ (Benítez-García et al., 2015).

**Fig. 1:**
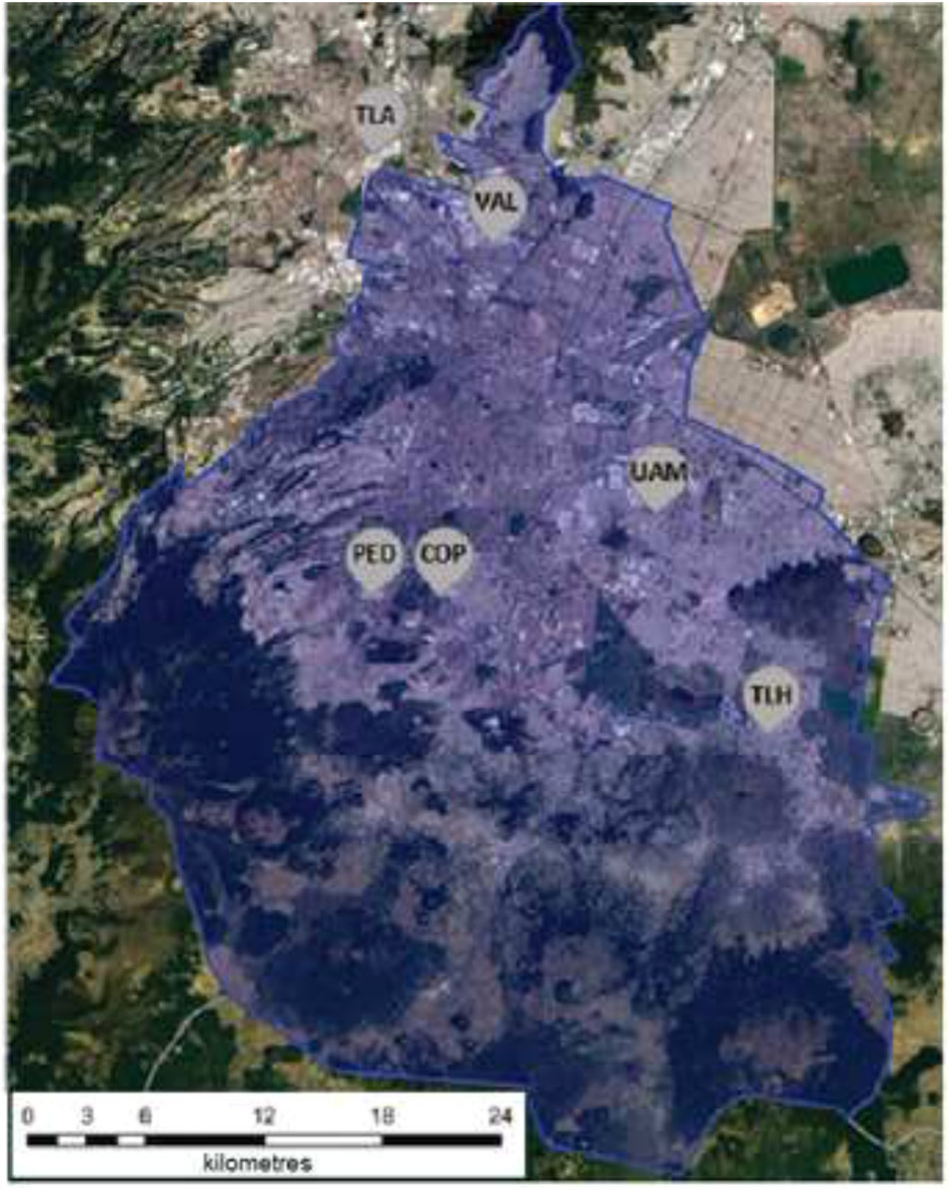
Location of our study sites in the Mexico City Metropolitan Area: Tlanepantla (TLA), Vallejo (VAL), Pedregal (PED), Cantera-Oriente Pedregal (COP), UAM-Iztapalapa (UAM), Tlahuac (TLH). Limits of Mexico City are delimited

Mexico City has an Air Quality Monitoring Network with a total of 32 automated stations for criteria gases and PM in MCMA. Monitoring data are transferred daily and hourly to an open access inventory (http://www.aire.cdmx.gob.mx). We selected 6 study sites from nearby these 32 stations to create a gradient of air pollution: Tlanepantla (TLA): 19°31′44.677′′N, 99°12′16.549′′O; Vallejo (VAL): 19°29′3.552′′N, 99°8'44.77′′O; Pedregal (PED): 19°19′30.526′′N, 99°12′14.889′′O; Cantera-Oriente Pedregal (COP): 19°19′12.565′′, 99°10'25.172′′O; UAM-Iztapalapa (UAM): 19°21′38.858′′N, 99°4′25.968′′O; and Tlahuac (TLH): 19°14′47.252′′N, 99°0′38.03′′O (Fig. 1). Four study sites are located within an automated station, and VAL and COP were located nearby stations (less than 4 km). The data for VAL was obtained from the Camarones station and the data for COP were from the Pedregal station. The atmospheric pollutants considered for our study were: PM_10,_ O_3,_ SO_2,_ CO, and NO_x_. We used the mean concentrations of each pollutant from 1 August 2013 to 31 May 2014 to determine our six sampling zones. This period includes the molting period of house sparrows, which occurs between August-September (personal observation JS), and the period in which immune response was assessed for each individual. In preliminary analyses, we also calculated the mean concentrations of each pollutant recorded during the molting period of sparrows (August and September 2013) for CORT analysis, and pollutants recorded during captures (the mean pollution record a month before each bird sampling) for the immunological analysis (February, March, and April 2014 depending on the capture of each individual). The results obtained with these mean concentrations showed similar relationships between study variables (data not shown). Given the particular climatic and topographic conditions of the city that may promote daytime variations in pollutants measurements, we think that analyzing a period of time from molt to sampling provides a more robust and representative measurement of the degree of pollution in each sampling zone, which can simultaneously relate to both physiological responses (stress and immune).

To conduct a better characterization of each of our study sites, and in order to understand the role of other anthropogenic variables that could also influence the physiology of the birds, we recorded the following variables at each site: Human population density (number of inhabitants/km^2^), housing density (number of houses/km^2^), industry density (number of industrial complexes/km^2^), and land-use (percentage of urban, agricultural and forest land). We obtained this information from the National Institute of Statistics and Geography of Mexico (INEGI: http://www.inegi.org.mx/) for each one of the municipalities where our study sites were located.

### Field sample collections

We captured house sparrows from March to May of 2014 (77 house sparrows; 33 males, and 44 females. Table 2) using mist nets. After sampling, the birds were released. All individuals were weighed using a digital balance (sensitivity 0.01 g), and the tarsus length was measured to the nearest 0.1 mm using a digital caliper. Body condition was quantified by sex as body mass relative to structural body size (tarsus length) by calculating the scaled mass index following Peig and Green (2010). We collected blood samples (150–200 μl) taken from the jugular vein. These were collected with heparinized syringes and kept chilled. On the day of collection, blood samples were centrifuged at 7000 *g* for 20 min to obtain plasma, which was frozen at −20 °C until physiological analysis.

### Stress response

Stress response was measured as the amount of corticosterone (CORT) deposited in the feathers, which provides a historical record of an individual’s hypothalamic–pituitary–adrenal (HPA) axis activity during the period of feather growth (Bortolotti et al., 2008). We collected the first rectrice from 70 individuals (31 males and 39 females). Feather corticosterone was determined using the method described by Bortolotti et al. (2008). The concentration of corticosterone in the feather extracts was measured with a radioimmunoassay using standard methods (see Blas et al. 2005). Feather corticosterone levels are expressed as a function of feather length (pg/mm). We validated this method for the recovery of exogenous CORT in house sparrow feathers by adding exogenous CORT to a pool of feather extracts and obtained a recovery rate of 92% (see Bortolotti et al. 2008 for details). Two types of results showed that our assays were suitable to represent stress levels: (1) feathers extracts in relation to the CORT standard curve showed similar slopes (CORT; *F*_3,12_ = 0.233; *p* = 0.329), and (2) the successful recovery of exogenous CORT added to control samples. We measured samples in three separate assays, with intra and inter-assay coefficients of variation of 3.72 and 2.30%.

### Innate immunity

Immunity was assessed following the assay described by Matson et al. (2005). This methodology allows for the simultaneous measurement of two constitutive innate immune functions: the hemagglutination reaction between natural antibodies and antigens (hereafter hemagglutination), and the hemolysis reaction of exogenous erythrocytes, which is a function of the number of lytic complement proteins present in the sampled blood (hereafter hemolysis). This is a non-invasive technique that requires a small volume of blood, does not involve the recapture of individuals, and is considered to be an integrative method to assess immunity (Matson et al., 2005; Palacios et al., 2012). Different studies have used this method to assess the costs and fitness consequences of immune responses (Møller and Haussy, 2007; Parejo and Silva, 2009; Nebel et al., 2012), to analyze immune responses under different ecological contexts, like a metal pollution gradient (Vermeulen et al. 2015), or under environmental heterogeneity (Pigeon et al., 2013), and to obtain an indicator of the study organisms’ health status (Deem et al., 2011).

The assay was conducted in 96-well round bottom assay plates (Corning Costar #3795). Twenty-five microliters of eight plasma samples were pipetted into columns 1 and 2 of the plate, and 25 µl of 0.01 M phosphate-buffered saline (PBS; Sigma #P3813, St Louis, MO) were added to columns 2–12. Using a multi-channel pipette, the contents of the column 2 wells were serially diluted (1:2) through column 11, resulting in dilutions ranging from 1 to 1/1024, with a total volume of 25 µl in every well. The 25 µl of PBS only in column 12 served as a negative control. For the assay itself, 25 µl of a 1% rabbit blood cell (HemoStats laboratories Dixon, CA, USA) suspension was added to all wells, effectively halving all plasma dilutions. Each plate was then sealed and gently vortexed for 10 s prior to incubation, during which time they were floated in a 37 °C water bath for 90 min. After incubation, plates were tilted at a 45° angle to their long axis for 20 min at room temperature, and then scanned to record the reaction of hemagglutination by natural antibodies. Plates were then kept at room temperature for an additional 70 min, and scanned for a second time to record complement-mediated maximum hemolysis. Quantification of hemagglutination and hemolysis was done by assessing the dilution stage (on a scale from 1 to 12) at which these two reactions stopped. It was not always possible to collect a blood sample from all captured individuals, therefore this analysis was conducted for samples from 59 individuals (27 males and 32 females).

### Data analyses

Hemolyisis, hemagglutination and CORT were not normally distributed (Shapiro-Wilk test; W < 0.63, *p* < 0.05). Although the hemolysis scores ranged from 0 to 4, this reaction was absent in 42 % of the individuals. Therefore, hemolysis scores were treated as a binary variable, i.e., ‘0’ (score 0; no hemolysis) or ‘1’ (score >0; lysis). Hemolysis and hemagglutination were not correlated (Spearman r = 0.182, *p* = 0.14, *n* = 66). Hemolysis and CORT were also not correlated (Spearman r = −0.084, *p* = 0.52, n = 60), nor was hemagglutination and CORT (Spearman r = −0.077, *p* = 0.56, *n* = 60). Therefore, these three variables were considered to be dependent variables in three independent models. A Principal Component Analysis (PCA) was performed on the 5 pollutants (PM_10,_ O_3,_ SO_2,_ CO, and NO_x_) for each one of our study sites. PCA is an analytic method that has been successfully used to determine Air Quality in recent decades (Smeyers-Verbeke et al., 1984; Chavent et al., 2008). PCA can identify relationships between the studied pollutants, and uncover patterns of air pollution (Voukantsis et al., 2011). The PC1 accounted for 83% of the total variance. PC1 was strongly positively related to 0_3_ concentration and strongly negatively related to the other four pollutants (Table 1). We used the values of the PC1 as an “ozone gradient” for our analyses. We performed another PCA with the six anthropogenic variables: Human population density, housing density, industry density, and land-use. The PC1 obtained in this analysis accounted for 54% of the total variance. PC1 scores were strongly positively related to human population, housing densities, and urban land. The scores close to zero were related to the density of industrial complexes. Lower scores in the PC1 were associated with a higher percentage of both agricultural and forest land (Table 1). As a result, we used the values of this PC1 as an “urban gradient”. We applied three generalized linear models (GzLM) with hemolysis (using a Binomial distribution and the complementary logit- link function), hemagglutination (using a Multinomial distribution and the complementary log- link function), and CORT (using a Normal distribution and the log link function) as dependent variables. Residuals were normally distributed after CORT was log-transformed (Shapiro-Wilk test; W = 0,98, *p* = 0.50). The three models included sex as a fixed factor and ozone gradient and urban gradient as covariates. We also included in each model the scaled mass index as a covariate to control for the potential effect of the body condition on the physiology, as well as all possible interactions between variables. The model selection was conducted by analyzing the distribution of our data and selecting for the models with the lowest AICc values (Akaike, 1974). Statistical analyses were performed using SPSS software (IBM SPSS Statistics 23, IBM Inc., NY, USA).

**Table 1:** Factors included in the principal component analyses

	PC1 O_3_ gradient		PC1 urban gradient
CO	−0.88	Agricultural land	−0.79
SO_*2*_	−0.91	Forest land	−0.29
PM_10_	−0.96	Industry density	0.01
NOx	−0.99	House density	0.83
O_3_	0.82	Urban land	0.93
		Human density	0.97
Percentage of variance	84		54

(1) ozone gradient from the five pollutant concentrations; and (2) urban gradient from the six variables of urbanization included in our study. The percentage of variance explained by each PC1 is shown at the end of the table.

## Results

**Fig. 2:**
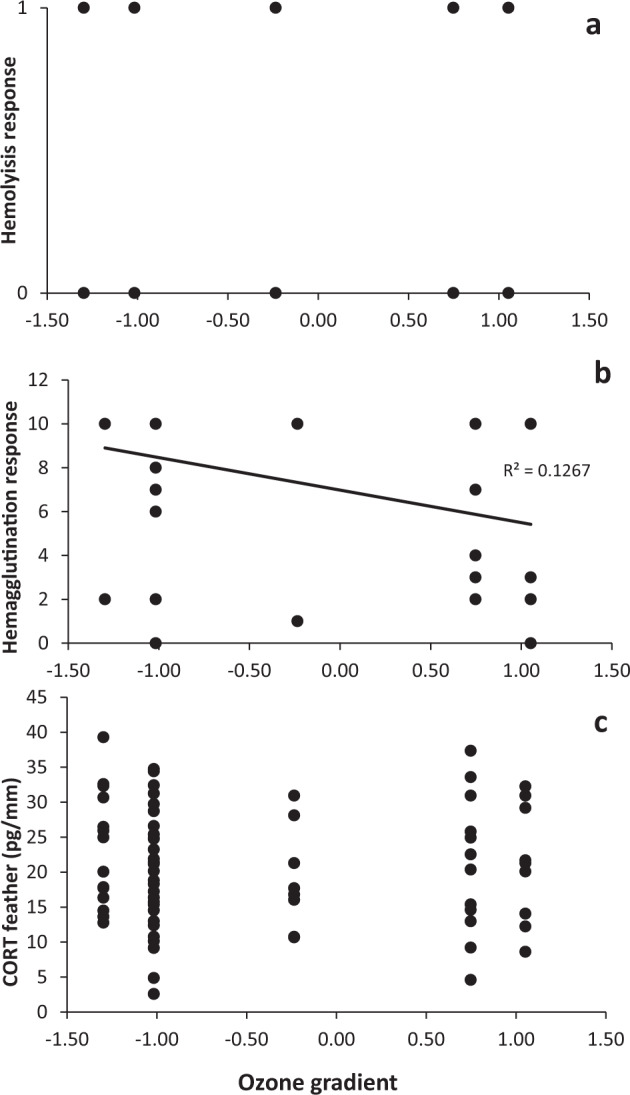
The relationship between: **A** Innate immunity as a function of the complement response of hemolysis to an ozone gradient; **B** Innate immunity as a function of the hemagglutination response of natural antibodies and ozone gradient; and **C** Feather corticosterone (CORT) and ozone gradient

Each study area was characterized by means of the two PCAs into both an ozone and an urban gradient. Tlahuac was the most ozone-contaminated but least urbanized study site. Tlaneplanta was the least ozone-contaminated area and Iztapalapa-UAM the most urbanized (Table 2). The Binomial Model showed that hemolysis did not relate to any of variables (Table 3; Fig. 2a.). GzLMs showed that hemagglutination was negatively related to the ozone gradient (Table 3; Fig. 2b.). However, the urban gradient, the scaled mass index, and sex were not related to this immune response. Finally, only sex was marginally related to the CORT concentration in feather; females had higher CORT in their feathers than males, although this difference was not significant (Table 3). The ozone gradient, the urban gradient, and the scaled mass index were also not related to the stress response (Table 3; Fig. 2c.). No interactions between variables were included in the three models with the lowest AICs (*p* > 0.05). Full models have been added as supplements (Supplements 1, 2, and 3), as well as a supplementary table (Supplement 4) with raw data and information on each house sparrow sampled.

**Table 2:** Mean values of the five air pollutant concentrations, six anthropogenic variables, and the two scores from both principal components analyses: PM_10_ (μg/m^3^), O_3_ (ppb), SO_2_ (ppb), CO (ppb), NOx (ppb)

	Site	Cantera-UNAM	Tlahuac	Iztapalapa-UAM	Vallejo	Tlanpantla	Pedregal
	*n* (males/ females)	5 (0/5)	11 (6/5)	10 (4/6)	30 (15/15)	16 (7/9)	5 (1/4)
OZONE GRADIENT	PM_10_ (SE)	40 (0, 27)	42 (0, 33)	48 (0, 30)	50 (0, 30)	53 (0, 32)	40 (0, 27)
	O_3_ (SE)	28 (0, 31)	32 (0, 32)	27 (0, 35)	23 (0, 31)	26 (0, 32)	28 (0, 31)
	SO_*2*_ (SE)	4 (0, 07)	3 (0, 04)	4 (0, 09)	7 (0, 15)	9 (0, 18)	4 (0, 07)
	CO (SE)	0, 5 (0, 004)	0, 6 (0, 005)	0, 9 (0, 007)	0, 8 (0, 007)	1, 0 (0, 007)	0, 5 (0, 004)
	NOx (SE)	38 (0, 33)	29 (0, 24)	52 (0, 5)	70 (0, 68)	68 (0, 63)	38 (0, 33)
	PC1	0.75	1.05	−0, 23	−1, 02	−1, 30	0, 75
URBAN GRADIENT	Human population density	11, 50	4, 20	16, 00	13, 50	8, 20	7, 60
	House density	3357	2342	4059	3641	2051	2058
	Industry density	1, 80	0, 80	4, 80	3, 25	28, 40	1, 36
	Urban land	100	34	96	100	91	74
	Agricultural land	0	66	4	0	9	0
	Forest land	0	0	0	0	0	26
	PC1	0, 58	−1, 60	1, 02	0, 81	−0, 22	−0, 59

Data obtained from the Air Quality Monitoring Network of Mexico City (1 August 2013–31 May 2014). Human population density (inhabitants/km^2^) × 10^3^, housing density (Houses/ km^2^), industry density (industries/km^2^) and urban land use (%), agricultural land use (%), and forest land (%). Data obtained from INEGI (2014 date of the consultation)

**Table 3:** Minimal adequate models showing the relationship between CORT concentration and sex, hemolysis response and ozone gradient, hemagglutination response, and sex

Response variable	Independent variables	Estimate	lower 95% CI	upper 95% CI	*X*^2^ Wald	*p*
**Hemolysis**	sex	−0.44	−1,50	0.62	0.665	0.415
**Hemagglutination**	ozone gradient	−0.72	−1.18	−0.25	9.126	**0.003**
**CORT**	sex	−0.19	−0.40	0.01	3.514	0.061

Bold values indicates statistical significant *p* values (*p* < 0.01)

## Discussion

In this study, we examined the influence of air pollution on the physiological stress (measured as CORT levels in feathers) and immune response (natural antibodies and complement system activity) of house sparrows living in MCMA. We did not find a significant influence of air pollution on the CORT concentrations or on the complement system activity of house sparrows. However, individuals captured at sites with a high concentration of ozone showed a reduction in natural antibody activity compared to those captured at sites with lower ozone concentrations. These results suggest that ozone concentrations in air pollution within MCMA may constrain the natural antibody response in the immune system of house sparrows.

As predicted, we found a negative relationship between air pollution and immune response in house sparrows. Exposure to ozone has been linked to adverse health effects in humans, including increased rates of visits to the hospital, exacerbation of chronic respiratory conditions (eg, asthma), decreased lung function, and increased mortality counts (Bell et al., 2004; Bell et al., 2007; Fann et al., 2015). Ozone increases the production of free radicals, which promotes a state of oxidative stress in eukaryotic aerobic organisms that is implicated in a wide variety of degenerative diseases (Kampa and Castanas, 2008). Several studies on mammals have shown functional, structural, and biochemical alterations caused by acute and/or chronic exposures to ozone concentrations (Dorado-Martínez et al., 2001; Valacchi et al., 2002; Wang et al., 2006; Ramot et al., 2015).

A recent study has shown large-scale evidence that air pollution, specifically ozone, is associated with declines in bird abundance in the United States (Liang et al., 2020). However, the effects of ozone on the physiology of birds is still poorly known. In general, ozone, as well other factors (e.g., temperature, ultraviolet light, mycotoxins, ammonia), is considered a potential avian stressor (Davison et al., 2008). However, few avian studies corroborate this hypothesis. An experimental study (Rombout et al., 1991) conducted on Japanese quails (*Coturnix japonica*) showed harmful effects of ozone on their lungs. Individuals continuously exposed to different ozone concentrations for 7 days showed a loss of cilia in the trachea and bronchi, an inflammatory response, necrosis of air capillary epithelial cells, and extensive hemorrhages, among other adverse effects. Since no signs of repair in the air capillary epithelium occurred after 7 days of continuous exposure, the authors concluded that quail seems to lack the morphological and biochemical ability to repair this tissue that has been observed in mammals (van Bree et al., 2001, but see: Gorriz et al., 1994). If this lack of repair capacity is found in more species, birds could be a group especially vulnerable to ozone. There is evidence that high concentrations of ozone have a negative effect on both adaptive and innate immune responses in mammals (reviewed in Jakab et al., 1995). In the case of the innate immune system, most of the studies assessing the effect of ozone are focused on macrophages and neutrophils, which are involved in phagocytosis of pathogens and inflammatory responses. These studies have shown that ozone reduces phagocytosis activity (Becker et al., 1991; Hollingsworth et al., 2007; Valentine, 1985) and impairs clearance in several species (Gilmour et al., 1993; Miller and Ehrlich, 1958). However, it should be noted that the pollutant concentrations to which the animals were subjected in these studies exceeded the concentrations the sparrows in our study were exposed to. Therefore, experimental studies at pollutant concentrations closer to natural conditions are needed to understand the true health risk faced by animals in the field.

The likelihood of humoral immunity perturbation by ozone is not known, especially with regard to naturally occurring antibodies (Nabs) and the complement system, which are components of the humoral innate immune response. Our study shows that individuals captured at sites with high levels of ozone had a diminished hemagglutination response. The hemagglutination response is measured to estimate the levels of circulating natural antibodies. A high hemagglutination response is related to high Nabs levels (Matson et al., 2005). Nabs are antibodies produced by B lymphocytes, essentially of the immunoglobulin M (IgM) isotype, although both immunoglobulins G (IgG) and A (IgA) isotypes have also been reported as Nabs (Panda and Ding, 2015). The production of IgGs and IgAs decreases in the presence of ozone, which has been shown in human B lymphocytes in vitro (Becker et al. 1991) and in mice (Gil-mour and Jakab, 1991). These results suggests that Nabs production could be limited by ozone exposure. However, experimental studies are necessary to corroborate our findings.

Natural antibodies activate the classical complement pathway leading to lysis, which reflects the interaction of complement and Nabs (Matson et al. 2005), if high ozone concentration decreases circulating Nabs levels, a decrease in the hemolysis response may also be expected. However, we did not find a relationship between ozone contamination and hemolysis. This result may reflect different fitness costs of maintaining Nabs and the complement system. Substantial nutritional and energetic costs are associated with maintenance of a normal immune system (Lochmiller and Deerenberg 2000). The development and maintenance of natural antibodies in birds is not entirely understood, but it is thought to require stimulation of an acquired immune system via B-1 cells by auto-antigens (Parmentier et al., 2004; Haghighi et al., 2006). This may be more costly than the development of defenses that depend on the innate immune system (i.e: complement system), a process that has been characterized as inexpensive (Lee 2006; Lee et al. 2008). Therefore, in adverse conditions, where the individual must face strong physiological trade-offs, it could be expected that the most costly response (i.e: Nabs response) is the most compromised.

Natural antibodies establish the first line of defense against invading pathogens, therefore an inefficient response of these components may be detrimental to the organisms (Matson et al., 2005). Lee and Klasing (2004) predicted that house sparrows (considered an invasive species and a good invader of new sites; Lee 2002) may have a weak systemic inflammatory response, but a stronger humoral response compared to a poor invader. This is because systemic inflammation is costly both metabolically and behaviorally, and a good invader requires high energy for growth and reproduction; adaptations which favor the capacity to invade (Klasing and Korver, 1997; Bonneaud et al., 2003). In addition to their in the defense against invading pathogens, Nabs also have a regulatory role in anti-inflammatory reactions, essential to avoid systemic diseases (Schwartz-Albiez et al., 2009). Therefore, by living in high ozone concentration areas where the Nabs response is limited, the ability for house sparrows to expand their population could be restricted. Ozone is unlikely to be the only cause of the diminished immune response in house sparrows, but it may contribute through indirect effects. Future studies evaluating the effects of ozone on humoral innate immunity are necessary to know the global effect of the pollutant on the immune system of this organism and other bird species. It is important to point out that the relationship between Nabs activity and ozone gradient found here cannot explain all of the variability observed in the hemagglutination response. Other factors which may also impact this response, but not considered in this study, include parasitic load, reproductive status, and/or age. These factors cause such high immune variability (e.g Christe et al., 2000; de Lope et al., 1998; Morales et al., 2004 for parasitic load; Nordling et al., 1998, Hanssen et al., 2005 for status reproductive; De Coster et al., 2010, Stambaugh et al., 2011 for age). Further experimental studies are necessary to elucidate the underlying mechanisms driving variability in the innate response. Additionally, although ozone does effect the house sparrow immune system, the mechanism for how this occurs is not obvious. Perhaps, it may be related to an acclimation process. Acclimation is a phenotypic response to an environmental challenge that may improve an organism’s ability to survive under severe environmental conditions (Hoffmann, 1995). Such responses occur in many organisms faced with different anthropogenic challenges (Yauk et al., 2001). As we have said above, the house sparrow is an invasive species and one of the reasons for its widespread success may be due precisely to its ability to acclimatize to anthropogenic environments, and it is very likely that the effect of the ozone on other species that are not so ubiquitous would be more dramatic.

Contrary to our expectations, we did not find that air pollution or the urban gradient had a significant influence on CORT concentrations in the house sparrow. This finding is supported by other studies on endocrine ecology in this species (Eeva et al., 2005; Fokidis et al., 2009; Bókony et al., 2012; Meillère et al., 2015), but not by work carried out in other avian species (e.g., see Fokidis et al., 2009; Zhang et al., 2011; Meillère et al., 2016;). The range of values recorded in this study for CORT feather concentration (∼3–39 pg CORT/mm feather), with the lowest values being thirteen times lower than the highest ones, is even higher than the range of values recorded by other studies that have reported avian stress using feather samples (Lattin et al., 2011; Legagneux et al., 2013; Will et al., 2014). Therefore, it seems that house sparrows included in our study may be stressed, however, this response is independent of both air pollution and urbanization gradients.

Several authors have concluded that the relationship between urbanization and CORT levels is inconsistent and species dependent, and impacted by life-history stage, age, sex, and the specific constraints of a given urban habitat (reviewed in Bonier, 2012). Sex, reproductive status, and food availability, could all have masked the effects of urbanization or air pollution on stress physiology (Dantzer et al., 2014). In fact, our results show marginally significant sexual differences in CORT, where females had higher CORT concentrations than males. Such difference could be caused by sex-specific costs of reproduction in house sparrows. In this species, females have an extra cost during reproduction, which could increase their CORT levels above those present in males. This hypothesis is supported by the findings of Bortolotti and colleagues (2008), who reported a positive relationship between CORT in feathers in the summer and fall, and a reproductive investment in eggs in the spring and summer for females of house sparrows this species. These authors suggest that elevated HPA activity during egg laying in female birds is costly, thus CORT levels found here could indicate that egg production is stressful, or that the current reproductive investment reduces the subsequent resistance to stress. It is noteworthy, that the exact period of molt is unknown for each individual in our study. We had to make certain assumptions about the timing of molts, though feathers grow at the same time when conditions are equivalent for individuals in the same place, for example in terms of food availability (Romero and Fairhurst, 2016). This together with sex distribution is not strictly homogeneus along our study area, and thus could be masking the effect of ozone pollution on stress level of house sparrows.

## Conclusion

Ozone is considered one of the most harmful constituents of the lower atmosphere because it acts as a major oxidizing agent (Alloway and Ayres, 1997). It is a green-house gas that affects the growth of plants (Krupa et al., 2001), causes adverse effects on human health (Kampa and Castanas 2008), and is related to the decline of bird populations (Liang et al., 2020). Despite numerous governmental campaigns focused on improving the air quality in the MCMA, ozone concentrations remain high and is the air pollutant that exceeds the environmental standards more days per year (more than 100 days/year; Benitez-Garcia et al., 2015; Jaimes-Palomera et al., 2016). The present study shows the potential impact of ozone pollution on a wild species in the MCMA for the first time, using Nabs activity as an indicator to assess the effect of air contamination on wildlife. This effect could be expected in more species. House sparrow populations are on the decline globally, and many causes have been suggested to explain this decline, including agricultural management (Wretenberg et al. 2006), habitat loss, and human influence (Hole et al., 2002, Shaw et al., 2008). Our results suggest that ozone may be a factor limiting the expansion of this species and may be playing a role in the observed population declines at large urban sites (Summers-Smith, 2003). Future studies in house sparrows and native species of Mexico should be performed to understand the noxious effects of ozone on the avian community. The results of such studies could be useful for managing bird populations inside polluted cities.

## Supplementary information

Supplement 1

Supplement 2

Supplement 3

Supplement 4
