# Supplementary material for: Physiological status of House Sparrows (Passer domesticus) along an ozone pollution gradient
Source: Ecotoxicology. 2023 Feb 21;32(2):261–72. doi: 10.1007/s10646-023-02632-z (PMC10008774; doi:10.1007/s10646-023-02632-z)
Supplement: Supplementary file 1 — Supplement 1 [file 10646_2023_2632_MOESM1_ESM.docx]

|  |  |  |  |  |  |  |
| --- | --- | --- | --- | --- | --- | --- |
| Response variable | Independent variables | Estimate | lower 95% CI | upper 95% CI | Wald | p |
|  |  |  |  |  |  |  |
| HEMOLYSIS | Intercept | 4,44 | -3,99 | 12,86 | 1,064 | 0,302 |
|  | sex=male | -10,24 | -23,87 | 3,39 | 2,169 | 0,141 |
|  | sex=female | 0 |  |  |  |  |
|  | scaled mass index | -0,22 | -0,56 | 0,13 | 1,500 | 0,221 |
|  | urban gradient | -4,31 | -15,50 | 6,87 | 0,572 | 0,450 |
|  | ozone gradient | -0,61 | -18,96 | 6,73 | 0,871 | 0,351 |
|  | sex=male*scaled mass index | 0,40 | -0,16 | 0,97 | 1,946 | 0,163 |
|  | sex=female*scaled mass index | 0 |  |  |  |  |
|  | sex=male*urban gradient | -0,27 | -1,99 | 1,44 | 0,100 | 0,752 |
|  | sex=female*urban gradient | 0 |  |  |  |  |
|  | sex=male*ozone gradient | -0,47 | -2,35 | 1,40 | 0,245 | 0,620 |
|  | sex=female*ozone gradient | 0 |  |  |  |  |
|  | scaled mass index*urban gradient | 0,19 | -0,29 | 0,66 | 0,574 | 0,449 |
|  | scaled mass index*ozone gradient | 0,26 | -0,28 | 0,81 | 0,898 | 0,343 |
|  | urban gradient*ozone gradient | -0,90 | -2,01 | 0,21 | 2,535 | 0,111 |
|  |  |  |  |  |  |  |
|  |  |  |  |  |  |  |
