# Supplementary material for: Physiological status of House Sparrows (Passer domesticus) along an ozone pollution gradient
Source: Ecotoxicology. 2023 Feb 21;32(2):261–72. doi: 10.1007/s10646-023-02632-z (PMC10008774; doi:10.1007/s10646-023-02632-z)
Supplement: Supplementary file 2 — Supplement 2 [file 10646_2023_2632_MOESM2_ESM.docx]

|  |  |  |  |  |  |  |  |  |
| --- | --- | --- | --- | --- | --- | --- | --- | --- |
| Response variable |  | Independent variables | Estimate | lower 95% CI | upper 95% CI | Wald | p |  |
|  |  |  |  |  |  |  |  |  |
| HEMAGGLUTINATION | Threshold | HEMA=0.00 | -4,05 | -9,17 | 1,09 | 2,388 | 0,122 |  |
|  |  | HEMA=1.00 | -3,85 | -8,97 | 1,27 | 2,177 | 0,140 |  |
|  |  | HEMA=2.00 | -3,41 | -8,52 | 1,69 | 1,720 | 0,190 |  |
|  |  | HEMA=3.00 | -2,68 | -7,75 | 2,40 | 1,070 | 0,301 |  |
|  |  | HEMA=4.00 | -2,59 | -7,66 | 2,48 | 1,000 | 0,317 |  |
|  |  | HEMA=6.00 | -2,50 | -7,56 | 2,56 | 0,935 | 0,333 |  |
|  |  | HEMA=7.00 | -2,41 | -7,46 | 2,64 | 0,876 | 0,349 |  |
|  |  | HEMA=8.00 | -2,33 | -7,38 | 2,71 | 0,822 | 0,365 |  |
|  |  | sex=male | -1,30 | -6,74 | 4,14 | 0,220 | 0,639 |  |
|  |  | sex=female | 0 |  |  |  |  |  |
|  |  | scaled mass index | -0,06 | -0,26 | 0,14 | 0,333 | 0,564 |  |
|  |  | urban gradient | -4,13 | -11,48 | 3,22 | 1,215 | 0,270 |  |
|  |  | ozone gradient | -6,52 | -16,57 | 3,54 | 1,612 | 0,204 |  |
|  |  | sex=male*scaled mass index | 0,09 | -0,13 | 0,31 | 0,626 | 0,429 |  |
|  |  | sex=female*scaled mass index | 0 |  |  |  |  |  |
|  |  | sex=male*urban gradient | 0,72 | -0,96 | 2,41 | 0,708 | 0,400 |  |
|  |  | sex=female*urban gradient | 0 |  |  |  |  |  |
|  |  | sex=male*ozone gradient | 0,89 | -1,16 | 2,90 | 0,740 | 0,390 |  |
|  |  | sex=female*ozone gradient | 0 |  |  |  |  |  |
|  |  | scaled mass index*urban gradient | 0,14 | -0,16 | 0,44 | 0,843 | 0,359 |  |
|  |  | scaled mass index*ozone gradient | 0,23 | -0,18 | 0,64 | 1,199 | 0,273 |  |
|  |  | urban gradient*ozone gradient | 0,73 | -0,32 | 1,76 | 1,868 | 0,172 |  |
|  |  |  |  |  |  |  |  |  |
|  |  |  |  |  |  |  |  |  |
