# Supplementary material for: Physiological status of House Sparrows (Passer domesticus) along an ozone pollution gradient
Source: Ecotoxicology. 2023 Feb 21;32(2):261–72. doi: 10.1007/s10646-023-02632-z (PMC10008774; doi:10.1007/s10646-023-02632-z)
Supplement: Supplementary file 3 — Supplement 3 [file 10646_2023_2632_MOESM3_ESM.docx]

|  |  |  |  |  |  |  |  |
| --- | --- | --- | --- | --- | --- | --- | --- |
| Response variable | Independent variables | Estimate | lower 95% CI | upper 95% CI | Wald | p |  |
|  |  |  |  |  |  |  |  |
| CORTICOSTERONE | Intercept | 3,20 | 1,83 | 4,58 | 20,896 | <0.001 |  |
|  | sex=male | 0,77 | -1,98 | 3,51 | 0,298 | 0,585 |  |
|  | sex=female | 0 |  |  |  |  |  |
|  | scaled mass index | 0,00 | -0,06 | 0,05 | 0,007 | 0,932 |  |
|  | urban gradient | 0,96 | -0,60 | 2,52 | 1,462 | 0,227 |  |
|  | ozone gradient | 0,41 | -0,94 | 1,75 | 0,349 | 0,555 |  |
|  | sex=male*scaled mass index | -0,03 | -0,15 | 0,08 | 0,351 | 0,554 |  |
|  | sex=female*scaled mass index | 0 |  |  |  |  |  |
|  | sex=male*urban gradient | -0,02 | -0,30 | 0,27 | 0,013 | 0,908 |  |
|  | sex=female*urban gradient | 0 |  |  |  |  |  |
|  | sex=male*ozone gradient | 0,23 | -0,07 | 0,53 | 2,215 | 0,137 |  |
|  | sex=female*ozone gradient | 0 |  |  |  |  |  |
|  | scaled mass index*urban gradient | -0,04 | -0,11 | 0,02 | 1,660 | 0,198 |  |
|  | scaled mass index*ozone gradient | -0,02 | -0,08 | 0,03 | 0,569 | 0,451 |  |
|  | urban gradient*ozone gradient | 0,11 | -0,06 | 0,28 | 1,696 | 0,193 |  |
|  |  |  |  |  |  |  |  |
|  |  |  |  |  |  |  |  |
