# Supplementary material for: Physiological status of House Sparrows (Passer domesticus) along an ozone pollution gradient
Source: Ecotoxicology. 2023 Feb 21;32(2):261–72. doi: 10.1007/s10646-023-02632-z (PMC10008774; doi:10.1007/s10646-023-02632-z)
Supplement: Supplementary file 4 — Supplement 4 [file 10646_2023_2632_MOESM4_ESM.docx]

Supplement 4

Raw data of each house sparrow sampled. Sites: Cantera-UNAM (COP), Tlahuac (TLH), Iztapalapa-UAM (UAM), Vallejo (VAL), Tlanepantla (TLA), Pedregal (PED). Tarsus length (mm). Mass (g). Body condition was quantified by sex as body mass relative to structural body size (tarsus length). Corticosterone levels are expressed as a function of feather length (pg/mm). HEMA: Quantification of hemagglutination was done by assessing the dilution stage (on a scale from 1 to 12). HEMO: Hemolysis scores were treated as a binary variable, i.e., ‘0’ (score 0; no hemolysis) or ‘1’ (score >0; hemolysis). Human: Human population density (inhabitants/km2) x 103, Housing: housing density (Houses/ km2), Industry (industries/km2) and Urban: urban land use (%), Agricult: agricultural land use (%) and Forest: forest land (%). Mean values of the five air pollutant concentrations, six anthropogenic variables, and the two scores from both principal components analyses: PM10 (μg/m3), O3 (ppb), SO2 (ppb), CO (ppb), NOx (ppb). Ozone gradient (PC1) from the five pollutant concentrations; urban gradient (PC1) from the six variables of urbanization included in our study.

| **Individual** | **Sex** | **Site** | **Tarsus** | **Mass** | **Scaled Mass Indexsex** | **CORT** | **HEMA** | **HEMO** | **Human** | **Housing** | **Industry** | **Urban** | **Agricult** | **Forest** | **PM_10_** | **O_3_** | **SO_2_** | **CO** | **NO_X_** | **urban gradient** | **ozone gradient** |
| --- | --- | --- | --- | --- | --- | --- | --- | --- | --- | --- | --- | --- | --- | --- | --- | --- | --- | --- | --- | --- | --- |
| 3PED | male | PED | 17,25 | . | . | 4,6 | . |  | 7,6 | 2058 | 1,36 | 74 | 0 | 26 | 40 | 28 | 4 | 0,5 | 38 | -0,59 | 0,75 |
| 11VAL | male | VAL | 19,8 | . | . | 10,1 | 10 | 0 | 13,5 | 3641 | 3,25 | 100 | 0 | 0 | 50 | 23 | 7 | 0,8 | 70 | 0,81 | -1,02 |
| 13VAL | male | VAL | 15,06 | 22,6 | 26,36 | . | 2 | 0 | 13,5 | 3641 | 3,25 | 100 | 0 | 0 | 50 | 23 | 7 | 0,8 | 70 | 0,81 | -1,02 |
| 20VAL | male | VAL | 18,43 | 24,3 | 24,16 | 10,8 | 10 | 1 | 13,5 | 3641 | 3,25 | 100 | 0 | 0 | 50 | 23 | 7 | 0,8 | 70 | 0,81 | -1,02 |
| 23VAL | male | VAL | 18,1 | 24,7 | 24,91 | 24,8 | 10 | 1 | 13,5 | 3641 | 3,25 | 100 | 0 | 0 | 50 | 23 | 7 | 0,8 | 70 | 0,81 | -1,02 |
| 25VAL | male | VAL | 18,16 | 23,6 | 23,74 | 23,2 | 10 | 1 | 13,5 | 3641 | 3,25 | 100 | 0 | 0 | 50 | 23 | 7 | 0,8 | 70 | 0,81 | -1,02 |
| 26VAL | male | VAL | 17,07 | 22,96 | 24,25 | 21,3 | 0 | 0 | 13,5 | 3641 | 3,25 | 100 | 0 | 0 | 50 | 23 | 7 | 0,8 | 70 | 0,81 | -1,02 |
| 28VAL | male | VAL | 18,1 | 24,7 | 24,91 | 21,4 | 10 | 1 | 13,5 | 3641 | 3,25 | 100 | 0 | 0 | 50 | 23 | 7 | 0,8 | 70 | 0,81 | -1,02 |
| 29VAL | male | VAL | 17,79 | . | . | 28,7 | 10 | 0 | 13,5 | 3641 | 3,25 | 100 | 0 | 0 | 50 | 23 | 7 | 0,8 | 70 | 0,81 | -1,02 |
| 32VAL | male | VAL | 17,52 | 23,66 | 24,48 | 20,1 | 10 | 1 | 13,5 | 3641 | 3,25 | 100 | 0 | 0 | 50 | 23 | 7 | 0,8 | 70 | 0,81 | -1,02 |
| 35VAL | male | VAL | 18,99 | 25,4 | 24,66 | 14,5 | 7 | 1 | 13,5 | 3641 | 3,25 | 100 | 0 | 0 | 50 | 23 | 7 | 0,8 | 70 | 0,81 | -1,02 |
| 36VAL | male | VAL | 19,14 | 25,28 | 24,39 | 29,7 | . |  | 13,5 | 3641 | 3,25 | 100 | 0 | 0 | 50 | 23 | 7 | 0,8 | 70 | 0,81 | -1,02 |
| 37VAL | male | VAL | 16,08 | 21,53 | 23,84 | 16,4 | 10 | 0 | 13,5 | 3641 | 3,25 | 100 | 0 | 0 | 50 | 23 | 7 | 0,8 | 70 | 0,81 | -1,02 |
| 38VAL | male | VAL | 18,65 | 23,95 | 23,59 | 2,6 | . |  | 13,5 | 3641 | 3,25 | 100 | 0 | 0 | 50 | 23 | 7 | 0,8 | 70 | 0,81 | -1,02 |
| 74TLH | male | TLH | 18,39 | 22,36 | 22,27 | 21,7 | . |  | 4,2 | 2342 | 0,8 | 34 | 66 | 0 | 42 | 32 | 3 | 0,6 | 29 | -1,60 | 1,05 |
| 77TLH | male | TLH | 19,37 | 27,85 | 26,62 | 12,2 | 3 | 1 | 4,2 | 2342 | 0,8 | 34 | 66 | 0 | 42 | 32 | 3 | 0,6 | 29 | -1,60 | 1,05 |
| 78TLH | male | TLH | 18,48 | 23,05 | 22,87 | 32,3 | 10 | 1 | 4,2 | 2342 | 0,8 | 34 | 66 | 0 | 42 | 32 | 3 | 0,6 | 29 | -1,60 | 1,05 |
| 81TLH | male | TLH | 20,54 | 23,4 | 21,36 | 21,3 | 0 | 0 | 4,2 | 2342 | 0,8 | 34 | 66 | 0 | 42 | 32 | 3 | 0,6 | 29 | -1,60 | 1,05 |
| 82TLH | male | TLH | 18,44 | 22,87 | 22,73 | 29,2 | 10 | 0 | 4,2 | 2342 | 0,8 | 34 | 66 | 0 | 42 | 32 | 3 | 0,6 | 29 | -1,60 | 1,05 |
| 85TLH | male | TLH | 15,29 | 21,86 | 25,19 | 20,1 | 10 | 1 | 4,2 | 2342 | 0,8 | 34 | 66 | 0 | 42 | 32 | 3 | 0,6 | 29 | -1,60 | 1,05 |
| 91UIZ | male | UIZ | 16,84 | 21,99 | 23,48 | 16,0 | 10 | 0 | 16 | 4059 | 4,8 | 96 | 4 | 0 | 48 | 28 | 4 | 0,9 | 52 | 1,02 | -0,23 |
| 92UIZ | male | UIZ | 20,55 | 25,2 | 22,99 | . | 10 | 1 | 16 | 4059 | 4,8 | 96 | 4 | 0 | 48 | 28 | 4 | 0,9 | 52 | 1,02 | -0,23 |
| 95UIZ | male | UIZ | 19,23 | 24,12 | 23,19 | 28,1 | 10 | 1 | 16 | 4059 | 4,8 | 96 | 4 | 0 | 48 | 28 | 4 | 0,9 | 52 | 1,02 | -0,23 |
| 100UIZ | male | UIZ | 17,73 | 22,88 | 23,45 | 10,7 | 10 | 1 | 16 | 4059 | 4,8 | 96 | 4 | 0 | 48 | 28 | 4 | 0,9 | 52 | 1,02 | -0,23 |
| 105VAL | male | VAL | 18,66 | 21,87 | 21,53 | 13,0 | 8 | 1 | 13,5 | 3641 | 3,25 | 100 | 0 | 0 | 50 | 23 | 7 | 0,8 | 70 | 0,81 | -1,02 |
| 111VAL | male | VAL | 19,7 | 26,2 | 24,71 | 9,2 | 10 | 0 | 13,5 | 3641 | 3,25 | 100 | 0 | 0 | 50 | 23 | 7 | 0,8 | 70 | 0,81 | -1,02 |
| 116TLA | male | TLA | 17,58 | 24,48 | 25,26 | 32,6 | 10 | 1 | 8,2 | 2051 | 28,4 | 91 | 9 | 0 | 53 | 26 | 9 | 1 | 68 | -0,22 | -1,30 |
| 118TLA | male | TLA | 17,77 | 21,55 | 22,05 | 12,8 | 10 | 1 | 8,2 | 2051 | 28,4 | 91 | 9 | 0 | 53 | 26 | 9 | 1 | 68 | -0,22 | -1,30 |
| 119TLA | male | TLA | 19,61 | 22,83 | 21,61 | 13,6 | 10 | 1 | 8,2 | 2051 | 28,4 | 91 | 9 | 0 | 53 | 26 | 9 | 1 | 68 | -0,22 | -1,30 |
| 120TLA | male | TLA | 17,51 | 22,78 | 23,58 | 17,8 | 10 | 1 | 8,2 | 2051 | 28,4 | 91 | 9 | 0 | 53 | 26 | 9 | 1 | 68 | -0,22 | -1,30 |
| 125TLA | male | TLA | 18,76 | 23,01 | 22,56 | 17,7 | 10 | 0 | 8,2 | 2051 | 28,4 | 91 | 9 | 0 | 53 | 26 | 9 | 1 | 68 | -0,22 | -1,30 |
| 127TLA | male | TLA | 20,12 | 25,51 | 23,66 | 16,3 | 10 | 1 | 8,2 | 2051 | 28,4 | 91 | 9 | 0 | 53 | 26 | 9 | 1 | 68 | -0,22 | -1,30 |
| 137TLA | male | TLA | 19,02 | 25,89 | 25,11 | 20,0 | 10 | 0 | 8,2 | 2051 | 28,4 | 91 | 9 | 0 | 53 | 26 | 9 | 1 | 68 | -0,22 | -1,30 |
| 1PED | female | PED | 17,46 | 25 | 28,59 | 15,4 | . |  | 7,6 | 2058 | 1,36 | 74 | 0 | 26 | 40 | 28 | 4 | 0,5 | 38 | -0,59 | 0,75 |
| 4PED | female | PED | 18,83 | 25 | 22,98 | 9,2 | 3 | 1 | 7,6 | 2058 | 1,36 | 74 | 0 | 26 | 40 | 28 | 4 | 0,5 | 38 | -0,59 | 0,75 |
| 6PED | female | PED | 18,19 | 22 | 22,35 | 30,9 | . |  | 7,6 | 2058 | 1,36 | 74 | 0 | 26 | 40 | 28 | 4 | 0,5 | 38 | -0,59 | 0,75 |
| 8PED | female | PED | 17,75 | 25 | 27,26 | 33,6 | 10 | 0 | 7,6 | 2058 | 1,36 | 74 | 0 | 26 | 40 | 28 | 4 | 0,5 | 38 | -0,59 | 0,75 |
| 15VAL | female | VAL | 18,01 | 21,35 | 22,32 | 31,2 | . |  | 13,5 | 3641 | 3,25 | 100 | 0 | 0 | 50 | 23 | 7 | 0,8 | 70 | 0,81 | -1,02 |
| 16VAL | female | VAL | 17,85 | 24,65 | 26,44 | 25,4 | 10 | 1 | 13,5 | 3641 | 3,25 | 100 | 0 | 0 | 50 | 23 | 7 | 0,8 | 70 | 0,81 | -1,02 |
| 17VAL | female | VAL | 17,21 | 20,16 | 24,03 | 15,4 | 10 | 1 | 13,5 | 3641 | 3,25 | 100 | 0 | 0 | 50 | 23 | 7 | 0,8 | 70 | 0,81 | -1,02 |
| 18VAL | female | VAL | 18,2 | 26,21 | 26,58 | 15,7 | 6 | 1 | 13,5 | 3641 | 3,25 | 100 | 0 | 0 | 50 | 23 | 7 | 0,8 | 70 | 0,81 | -1,02 |
| 21VAL | female | VAL | 19,38 | 22,69 | 19,19 | 21,9 | 10 | 0 | 13,5 | 3641 | 3,25 | 100 | 0 | 0 | 50 | 23 | 7 | 0,8 | 70 | 0,81 | -1,02 |
| 22VAL | female | VAL | 19 | 22,49 | 20,14 | 32,4 | 10 | 0 | 13,5 | 3641 | 3,25 | 100 | 0 | 0 | 50 | 23 | 7 | 0,8 | 70 | 0,81 | -1,02 |
| 24VAL | female | VAL | 18,09 | . | . | 24,8 | 10 | 1 | 13,5 | 3641 | 3,25 | 100 | 0 | 0 | 50 | 23 | 7 | 0,8 | 70 | 0,81 | -1,02 |
| 27VAL | female | VAL | 17,52 | . | . | 18,3 | . |  | 13,5 | 3641 | 3,25 | 100 | 0 | 0 | 50 | 23 | 7 | 0,8 | 70 | 0,81 | -1,02 |
| 30VAL | female | VAL | 18,54 | 20,7 | 19,90 | 34,7 | 0 | 0 | 13,5 | 3641 | 3,25 | 100 | 0 | 0 | 50 | 23 | 7 | 0,8 | 70 | 0,81 | -1,02 |
| 31VAL | female | VAL | 16,8 | 18,34 | 23,44 | 4,9 | . |  | 13,5 | 3641 | 3,25 | 100 | 0 | 0 | 50 | 23 | 7 | 0,8 | 70 | 0,81 | -1,02 |
| 33VAL | female | VAL | 18,14 | 21,2 | 21,71 | 12,4 | 0 | 0 | 13,5 | 3641 | 3,25 | 100 | 0 | 0 | 50 | 23 | 7 | 0,8 | 70 | 0,81 | -1,02 |
| 34VAL | female | VAL | 19,16 | 22,87 | 19,99 | 34,4 | 0 | 0 | 13,5 | 3641 | 3,25 | 100 | 0 | 0 | 50 | 23 | 7 | 0,8 | 70 | 0,81 | -1,02 |
| 56COP | female | COP | 16,9 | 23,39 | 29,39 | 22,5 | . |  | 11,5 | 3357 | 1,8 | 100 | 0 | 0 | 40 | 28 | 4 | 0,5 | 38 | 0,58 | 0,75 |
| 57COP | female | COP | 18,33 | 23,7 | 23,55 | 14,6 | 3 | 0 | 11,5 | 3357 | 1,8 | 100 | 0 | 0 | 40 | 28 | 4 | 0,5 | 38 | 0,58 | 0,75 |
| 64COP | female | COP | 17,17 | 21,3 | 25,56 | 37,4 | 4 | 1 | 11,5 | 3357 | 1,8 | 100 | 0 | 0 | 40 | 28 | 4 | 0,5 | 38 | 0,58 | 0,75 |
| 65COP | female | COP | 18,26 | 24,8 | 24,91 | 13,0 | 3 | 1 | 11,5 | 3357 | 1,8 | 100 | 0 | 0 | 40 | 28 | 4 | 0,5 | 38 | 0,58 | 0,75 |
| 68COP | female | COP | 18,21 | 25,87 | 26,20 | 20,4 | . |  | 11,5 | 3357 | 1,8 | 100 | 0 | 0 | 40 | 28 | 4 | 0,5 | 38 | 0,58 | 0,75 |
| 70TLH | female | TLH | 18,22 | 22,9 | 23,15 | . | 2 | 1 | 4,2 | 2342 | 0,8 | 34 | 66 | 0 | 42 | 32 | 3 | 0,6 | 29 | -1,60 | 1,05 |
| 72TLH | female | TLH | 17,1 | 22,76 | 27,64 | 30,9 | 3 | 0 | 4,2 | 2342 | 0,8 | 34 | 66 | 0 | 42 | 32 | 3 | 0,6 | 29 | -1,60 | 1,05 |
| 73TLH | female | TLH | 15,71 | 22,8 | 35,38 | . | 3 | 1 | 4,2 | 2342 | 0,8 | 34 | 66 | 0 | 42 | 32 | 3 | 0,6 | 29 | -1,60 | 1,05 |
| 80TLH | female | TLH | 18,88 | 26,42 | 24,10 | 8,6 | . |  | 4,2 | 2342 | 0,8 | 34 | 66 | 0 | 42 | 32 | 3 | 0,6 | 29 | -1,60 | 1,05 |
| 87TLH | female | TLH | 18,34 | 22,78 | 22,60 | 14,1 | 10 | 0 | 4,2 | 2342 | 0,8 | 34 | 66 | 0 | 42 | 32 | 3 | 0,6 | 29 | -1,60 | 1,05 |
| 93UIZ | female | UIZ | 19,65 | . | . | 16,8 | 10 | 1 | 16 | 4059 | 4,8 | 96 | 4 | 0 | 48 | 28 | 4 | 0,9 | 52 | 1,02 | -0,23 |
| 94UIZ | female | UIZ | 19,08 | 23,58 | 20,86 | . | 10 | 1 | 16 | 4059 | 4,8 | 96 | 4 | 0 | 48 | 28 | 4 | 0,9 | 52 | 1,02 | -0,23 |
| 98UIZ | female | UIZ | 19,92 | 25,75 | 20,12 | 21,3 | 10 | 1 | 16 | 4059 | 4,8 | 96 | 4 | 0 | 48 | 28 | 4 | 0,9 | 52 | 1,02 | -0,23 |
| 99UIZ | female | UIZ | 19,82 | 29,08 | 23,05 | 30,9 | 1 | 0 | 16 | 4059 | 4,8 | 96 | 4 | 0 | 48 | 28 | 4 | 0,9 | 52 | 1,02 | -0,23 |
| 101UIZ | female | UIZ | 18,92 | 27,35 | 24,80 | 10,7 | 10 | 1 | 16 | 4059 | 4,8 | 96 | 4 | 0 | 48 | 28 | 4 | 0,9 | 52 | 1,02 | -0,23 |
| 104UIZ | female | UIZ | 17,5 | . | . | 17,7 | 10 | 0 | 16 | 4059 | 4,8 | 96 | 4 | 0 | 48 | 28 | 4 | 0,9 | 52 | 1,02 | -0,23 |
| 107VAL | female | VAL | 18,45 | 24,16 | 23,56 | 18,8 | 10 | 0 | 13,5 | 3641 | 3,25 | 100 | 0 | 0 | 50 | 23 | 7 | 0,8 | 70 | 0,81 | -1,02 |
| 108VAL | female | VAL | 19,03 | 25,56 | 22,79 | 26,6 | 10 | 1 | 13,5 | 3641 | 3,25 | 100 | 0 | 0 | 50 | 23 | 7 | 0,8 | 70 | 0,81 | -1,02 |
| 115VAL | female | VAL | 18,17 | 23,7 | 24,15 | 21,2 | 10 | 0 | 13,5 | 3641 | 3,25 | 100 | 0 | 0 | 50 | 23 | 7 | 0,8 | 70 | 0,81 | -1,02 |
| 117TLA | female | TLA | 19 | 25,22 | 22,59 | . | . |  | 8,2 | 2051 | 28,4 | 91 | 9 | 0 | 53 | 26 | 9 | 1 | 68 | -0,22 | -1,30 |
| 122TLA | female | TLA | 18,81 | 23,66 | 21,82 | 30,7 | 10 | 1 | 8,2 | 2051 | 28,4 | 91 | 9 | 0 | 53 | 26 | 9 | 1 | 68 | -0,22 | -1,30 |
| 124TLA | female | TLA | 19,21 | 24,71 | 21,44 | 25,0 | 10 | 1 | 8,2 | 2051 | 28,4 | 91 | 9 | 0 | 53 | 26 | 9 | 1 | 68 | -0,22 | -1,30 |
| 126TLA | female | TLA | 18,78 | 24,21 | 22,43 | 32,3 | 10 | 1 | 8,2 | 2051 | 28,4 | 91 | 9 | 0 | 53 | 26 | 9 | 1 | 68 | -0,22 | -1,30 |
| 128TLA | female | TLA | 18,32 | 24,19 | 24,07 | . | 10 | 1 | 8,2 | 2051 | 28,4 | 91 | 9 | 0 | 53 | 26 | 9 | 1 | 68 | -0,22 | -1,30 |
| 131TLA | female | TLA | 17,91 | 23,42 | 24,88 | 25,9 | 10 | 1 | 8,2 | 2051 | 28,4 | 91 | 9 | 0 | 53 | 26 | 9 | 1 | 68 | -0,22 | -1,30 |
| 132TLA | female | TLA | 17,06 | 23,88 | 29,20 | 39,3 | 2 | 1 | 8,2 | 2051 | 28,4 | 91 | 9 | 0 | 53 | 26 | 9 | 1 | 68 | -0,22 | -1,30 |
| 134TLA | female | TLA | 19,71 | 29,14 | 23,47 | 26,5 | 10 | 1 | 8,2 | 2051 | 28,4 | 91 | 9 | 0 | 53 | 26 | 9 | 1 | 68 | -0,22 | -1,30 |
| 135TLA | female | TLA | 18,13 | 23,51 | 24,11 | 14,5 | 10 | 0 | 8,2 | 2051 | 28,4 | 91 | 9 | 0 | 53 | 26 | 9 | 1 | 68 | -0,22 | -1,30 |
